# Transmission Dynamics of a Mycobacterium tuberculosis Complex Outbreak in an Indigenous Population in the Colombian Amazon Region

**DOI:** 10.1128/spectrum.05013-22

**Published:** 2023-05-24

**Authors:** Francy J. Pérez-Llanos, Viola Dreyer, Ivan Barilar, Christian Utpatel, Thomas A. Kohl, Martha Isabel Murcia, Susanne Homolka, Matthias Merker, Stefan Niemann

**Affiliations:** a Molecular and Experimental Mycobacteriology, Research Center Borstel, Borstel, Germany; b German Center for Infection Research, Hamburg-Lübeck-Borstel-Riems, Germany; c Grupo MICOBAC-UN, Departamento de Microbiología, Facultad de Medicina, Universidad Nacional de Colombia, Bogotá, Colombia; d Evolution of the Resistome, Research Center Borstel, Borstel, Germany; Yale University

**Keywords:** Whole-genome sequencing, Colombia, tuberculosis

## Abstract

Whole genome sequencing (WGS) has become the main tool for studying the transmission of Mycobacterium tuberculosis complex (MTBC) strains; however, the clonal expansion of one strain often limits its application in local MTBC outbreaks. The use of an alternative reference genome and the inclusion of repetitive regions in the analysis could potentially increase the resolution, but the added value has not yet been defined. Here, we leveraged short and long WGS read data of a previously reported MTBC outbreak in the Colombian Amazon Region to analyze possible transmission chains among 74 patients in the indigenous setting of Puerto Nariño (March to October 2016). In total, 90.5% (67/74) of the patients were infected with one distinct MTBC strain belonging to lineage 4.3.3. Employing a reference genome from an outbreak strain and highly confident single nucleotide polymorphisms (SNPs) in repetitive genomic regions, e.g., the proline-glutamic acid/proline-proline-glutamic-acid (PE/PPE) gene family, increased the phylogenetic resolution compared to a classical H37Rv reference mapping approach. Specifically, the number of differentiating SNPs increased from 890 to 1,094, which resulted in a more granular transmission network as judged by an increasing number of individual nodes in a maximum parsimony tree, i.e., 5 versus 9 nodes. We also found in 29.9% (20/67) of the outbreak isolates, heterogenous alleles at phylogenetically informative sites, suggesting that these patients are infected with more than one clone. In conclusion, customized SNP calling thresholds and employment of a local reference genome for a mapping approach can improve the phylogenetic resolution in highly clonal MTBC populations and help elucidate within-host MTBC diversity.

**IMPORTANCE** The Colombian Amazon around Puerto Nariño has a high tuberculosis burden with a prevalence of 1,267/100,000 people in 2016. Recently, an outbreak of Mycobacterium tuberculosis complex (MTBC) bacteria among the indigenous populations was identified with classical MTBC genotyping methods. Here, we employed a whole-genome sequencing-based outbreak investigation in order to improve the phylogenetic resolution and gain new insights into the transmission dynamics in this remote Colombian Amazon Region. The inclusion of well-supported single nucleotide polymorphisms in repetitive regions and a *de novo*-assembled local reference genome provided a more granular picture of the circulating outbreak strain and revealed new transmission chains. Multiple patients from different settlements were possibly infected with at least two different clones in this high-incidence setting. Thus, our results have the potential to improve molecular surveillance studies in other high-burden settings, especially regions with few clonal multidrug-resistant (MDR) MTBC lineages/clades.

## INTRODUCTION

Tuberculosis (TB) is one of the leading contagious diseases worldwide, with an estimated 10 million cases in 2020, 3% of which were in Latin America (LATAM) (1–3). In Colombia, an incidence of 27.69 cases per 100,000 inhabitants was reported (4, 5). Although drug resistance in LATAM affects mainly Peru (29.9%), Brazil (22.7%), Mexico (8.6%), and Colombia (5.3%), spillover effects to other countries are likely within the current antibiotic resistance crisis (6, 7).

In recent years, TB incidence in the WHO/Pan American Health Organization (PAHO) region of the Americas has been on the rise (3), especially in the indigenous population of LATAM, with nine times more burden than in the general population, with 11,608 cases reported per 100,000 indigenous people in 2018, 5.4% of which were from Colombia, particularly in communities of the Amazon (2). Members of these communities are at high risk of developing the disease given the isolated location and health, sociological, and economic inequalities that have hindered timely access to medical services (8, 9). In addition, the ongoing Coronavirus disease 2019 (COVID-19) pandemic has potentially worsened their situation and is therefore threatening public health efforts to end TB by 2035 (2, 3, 10, 11).

To advance faster in the control and elimination of the disease in these vulnerable populations, the World Health Organization (WHO) has endorsed whole-genome sequencing (WGS) as a tool for understanding local epidemics to enable better public health interventions (12, 13). WGS analyses allow for the rapid analysis of the genomes of clinical MTBC strains, enabling genome comparisons with high resolution, e.g., for outbreak analysis, reliable strain genotyping, drug resistance prediction, and identification of transmission chains (14–16). While MTBC genomic surveillance is already implemented in developed countries (17, 18), it is poorly integrated into the public health practices of LATAM due to high initial investments, equipment restrictions, and lack of expertise (14).

To better understand the spread of an MTBC outbreak that occurred in 2016 among 23 indigenous settlements of the Ticuna, Cocama, and Yagua ethnicities in the remote setting of Puerto Nariño, in the Colombian Amazon Region (19), we performed a WGS analysis of 74 MTBC isolates obtained from March to October 2016. This outbreak led to a reported TB prevalence of 1,267/100,000 inhabitants, which raised concerns among national and international public health authorities (20). Due to the low genetic diversity of the circulating strain, the outbreak could not be fully unraveled with the conventional molecular epidemiology methods available in the country (21). Aiming for an increased phylogenetic resolution and an improved understanding of the transmission dynamics in this indigenous and remote patient population, we investigated the outbreak isolates with a standard WGS approach and extended the analytical range to repetitive regions, low frequency variant detection, and mapping on a newly established “outbreak genome.”

## RESULTS

### Demographic characteristics of the populations studied.

**FIG 1:**
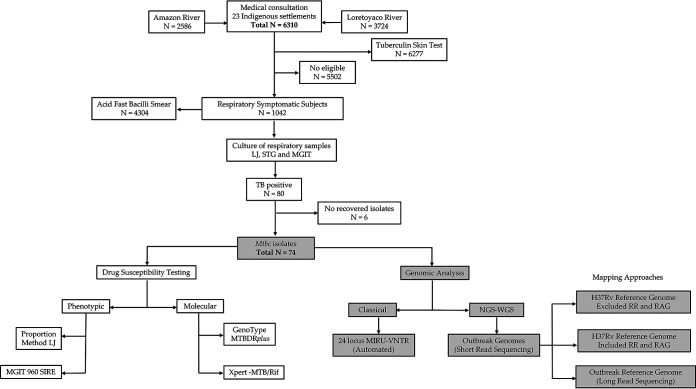
General workflow of the TB investigation in the remote setting of Puerto Nariño, Amazonas, Colombia. Gray highlighted squares point to the genomic investigation of this study. TB, tuberculosis; LJ, Löwenstein-Jensen; MGIT, mycobacterial growth indicator tube; MTBC, Mycobacterium tuberculosis complex; SIRE, streptomycin isoniazid rifampicin ethambutol; MTBDR, Mycobacterium tuberculosis drug resistance; MIRU-VNTR, mycobacterial interspersed repetitive-unit–variable-number tandem-repeats; NGS, next-generation sequencing; WGS, whole-genome sequencing; RR, repetitive regions; DRAG, drug resistance-associated genes.

**FIG 2:**
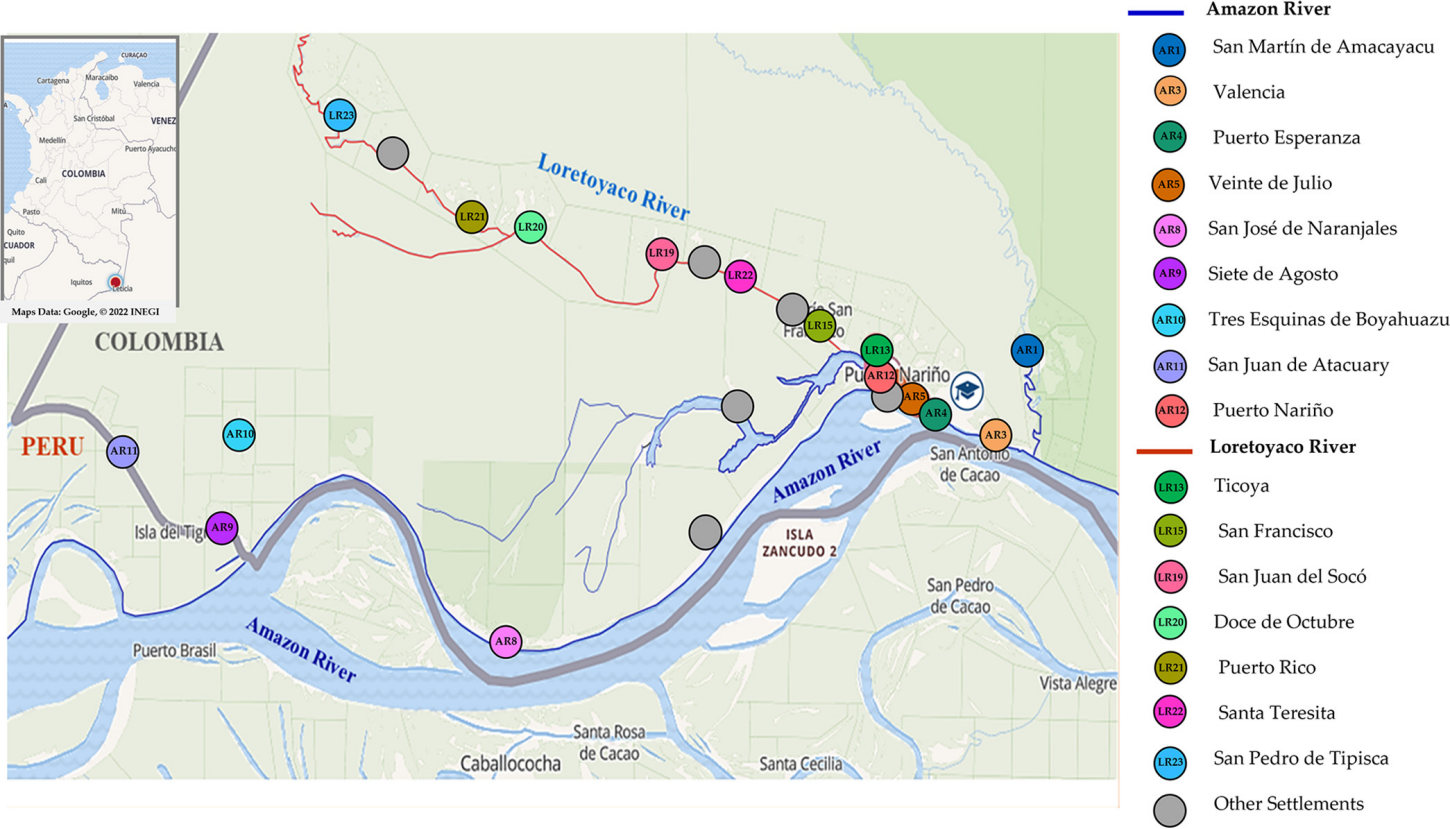
Geographical representation of the 16 indigenous settlements based in Puerto Nariño, Amazonas, Colombia. Colored circles indicate individual settlements on the shore of the Loretoyaco (red line) and the Amazon River (blue line). AR, Amazon River; LT, Loretoyaco River.

The TB outbreak comprised 80 positive cases out of 6,310 individuals screened from March to October 2016 (19), from which only 74 MTBC isolates (one isolate per patient) were recovered, and 70 of the patients self-identified as indigenous. These patients were inhabiting 16 indigenous settlements on the shore of the Loretoyaco and Amazon rivers (Fig. 1 and 2). The median age of the patients was 23.5 years (interquartile range, 9 to 51 years), 45/74 (60.8%) patients were male, and 31/74 (41.9%) patients were children (<16 years) (Table 1). Half of the patient population belonged to the Ticuna ethnicity, 64/74 (86.5%) patients were covered by the subsidized program, and 31/74 (41.9%) patients had no education. Risk factors of TB were identified, such as malnutrition in 26/74 (35.1%) patients and overcrowding conditions (8 to 14 people in a household) in 34/74 (45.9%) patients. Almost all patients (73/74; 98.6%) had at least one of the following TB symptoms: coughing, hemoptysis, weight loss, thoracic pain, fever, or asthenia.

**TABLE 1:** Demographic and clinical characteristics of 74 patients from 16 indigenous settlements in Puerto Nariño^*a*^

Demographic or clinical characteristic	No. of cases (%)^*b*^
Georeferencing	
Amazon River	31 (41.89)
Loretoyaco River	43 (58.1)
Detection case	
Primary consultation	62 (84)
Contact tracing	12 (16,2)
Gender	
Male	45 (60.8)
Female	29 (39.2)
Age categories	
Child:1–15 yrs	31 (41.9)
Youth: 16–26 yrs	7 (9.5)
Adult: 27–59 yrs	27 (36,4)
Old adult: ≥60 yrs	9 (12,16)
Insurance type	
Not insured	6 (8.1)
Subsidized	64 (86.5)
Employer-based	3 (4.1)
Other	1 (1.4)
Schooling level	
No education	31 (41.89)
Primary	43 (58,10%)
Secondary incomplete	14 (18.9)
Indigenous ethnicity	
Ticuna	37 (50)
Cocama	14 (18.9)
Yagua	19 (25.7)
No Indigenous	4 (5.4)
TB risk factors and comorbidities	
Human immunodeficiency virus unknown	64 (86.5)
Drug use	1 (1.4)
Alcohol	13 (17.6)
Tobacco Smoking	9 (12.2)
Malnutrition^*c*^	26 (35.1)
Other medical diagnoses	5 (6.8)
Overcrowding conditions^*d*^	
Household of 2–7 people	40 (54.1)
Household of 8–14 people	34 (45.9)
Bacillus Calmette-Guérin vaccination scar	
Presence	47 (63.5)
Absence	27 (36.5)
Tuberculin skin test	
Positive	28 (37.8)
Negative	34 (45.9)
Unknown	12
Previous TB diagnosis and Treatment	
Reported	6 (8.1)
Not reported	68 (91.9)
Recent TB contact	
Reported	26 (35.1)
Not reported	48 (64.9)
Symptoms of TB^*e*^	
Presence	73 (98.6)
Absence	1 (1.4)
Pulmonary aggregates	
Presence	13 (17.6)
Absence	61 (80)
Acid fast bacilli smear	
Positive	73 (98.8)
Negative	1 (1.4)
Drug sensitivity, rifampicin-isoniazid	
Sensitive	73 (98.6)
Resistance	1 (1.4)

a Data are depicted in the table as absolute numbers followed by the percentages (%), which were calculated based on the number of people for a given characteristic out of the total number of 74 TB cases with a single MTBC isolate of the Puerto Nariño outbreak. Percentages were rounded and may not total 100.

b Percentages were calculated based on a total number of 74 patients (100%).

c Malnutrition was defined as a body mass index (BMI) of <18 kg/m^2^.

d Household crowding was defined as two or more people were living in the same house.

e TB symptoms such as coughing of any duration, hemoptysis, weight loss, thoracic pain, fever, asthenia, and others. Abnormal respiratory sounds: rhonchus, coarse crackles, bronchial breath, or rales over upper lobes or other regions from both lung fields.

### Classical genotyping of the LAM-MTBC outbreak.

Initially, we defined the genotypes of all 74 isolates with 24-loci mycobacterial interspersed repetitive-unit–variable-number tandem-repeat (MIRU-VNTR) typing. The MTBC population structure was found to be highly homogeneous; 74/74 (100%) of the isolates belonged to the Euro American lineage, also known as lineage 4 (L4), 72/74 of which (97.2%) were Latin American Mediterranean (LAM), and 2/74 (2.7%) were identified as the Haarlem genotype. Of the LAM isolates, 31/72 (43.05%) and 41/72 (56.94%) were from indigenous settlements based on the Amazon and Loretoyaco Rivers, respectively (see Table S1 in the supplemental material). One large cluster comprised 49 patients with identical 24-loci MIRU-VNTR patterns, including 16 children. A smaller cluster was observed with 11 patients and 2 alleles distances. Patients in both clusters originated from different settlements in the study region, and 8/74 (10.8%) isolates had mixed MIRU-VNTR alleles (Fig. S1A and B).

### Whole-genome sequencing analysis.

**FIG 3:**
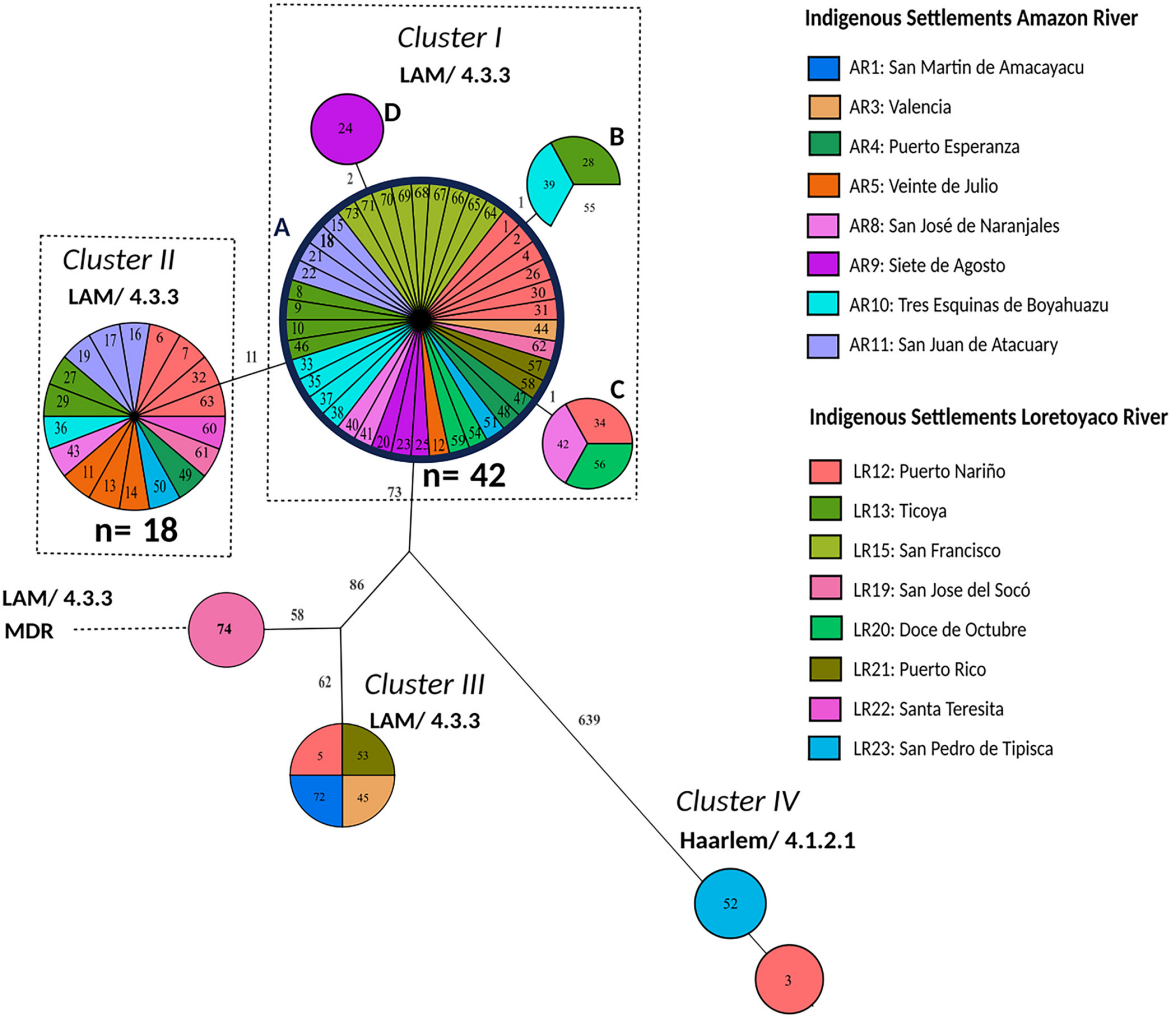
Genetic relationships of patient isolates based on an H37Rv reference mapping approach. Isolates were sampled from March to October 2016 in Puerto Nariño, Amazonas, Colombia. The maximum parsimony tree (MPT) was based on 890 concatenated single nucleotide polymorphisms (SNP) of 74 Mycobacterium tuberculosis complex (MTBC) isolates recovered from 16 indigenous settlements. The size of each node is proportional to the number of isolates. The genetic distance is indicated on branches as the number of SNPs that differ between nodes. Samples are color-coded based on the patient’s settlement and identified with an ID number. In the data sets, the sample ID number is preceded by the label COL, which refers to Colombia. Outbreak clusters are named I, II, III, and IV. Clusters comprising the clonal events are shown with dashed boxes, and nodes of cluster I are termed A, B, C, and D. LAM, Latin American Mediterranean; MDR, multidrug-resistant; AR, Amazon River; LR, Loretoyaco River.

WGS was successfully performed for all 74 MTBC isolates. Phylogenetic classification of the isolates based on specific single nucleotide polymorphism (SNP) signatures described previously by Coll et al. classified them into 72 LAM sublineage 4.3.3., and two Haarlem sublineage 4.1.2.1 isolates (22) (Table S1). Following an H37Rv reference-mapping approach allowed us to build a maximum parsimony tree (MPT) based on the SNP distance matrix of 890 SNPs, which confirmed the clonal population structure of the outbreak. Cluster analysis revealed two main molecular transmission chains of LAM 4.3.3. strains named cluster I and cluster II (Fig. 3). Furthermore, two other clusters were noted, i.e., cluster III, comprising four LAM 4.3.3. strains separated by more than 200 SNPs from cluster I/II, and cluster IV, comprising two Haarlem strains (Fig. 3).

The largest cluster, cluster I, contained 42 identical (42/74, 56.8%) isolates and 7 isolates differing by a maximum of two SNPs (Fig. 3). Out of the 49 patients in this largest transmission network, 20 were children from distinct settlements and river locations (Fig. S2 and S3 and Table S1). The second cluster, cluster II comprised 18 identical (18/74, 24.3%) isolates, with 11 SNPs separating both clusters. This suggests a relatively recent common ancestor; however, the clear separation by 11 SNPs indicates two independent short-term transmission chains in the study region.

Molecular drug resistance prediction detected multidrug resistance in one LAM/4.3.3 isolate from a child (COL-74), which had mutations mediating resistance to all first-line drugs. Although this case was identified by contact tracing, it was not in a molecular cluster (Fig. 3). The resistance-mediating mutations were *rpoB* Ser450Leu (99.64%), *katG* Ser315Thr (100%), *pncA* His82Asp (99.68%), *embB* Met306lle (100%), and *rpsL* Lys88Thr (100%) conferring resistance to rifampicin, isoniazid, pyrazinamide, ethambutol, and streptomycin, respectively (Fig. 3). In addition, we found resistance-associated SNPs with a low allele frequency in two isolates/patients (LAM 4.3.3/COL-18 and Haarlem 4.1.2.1/COL-3), i.e., *rpoB* Ser431Thr (6.62%) and *rpsA* Thr210Ala (8.21%) in COL-18, and *rpsA* Thr5Ala (10.57%) in COL-3.

### Extended WGS analysis of the LAM-MTBC outbreak.

**FIG 4:**
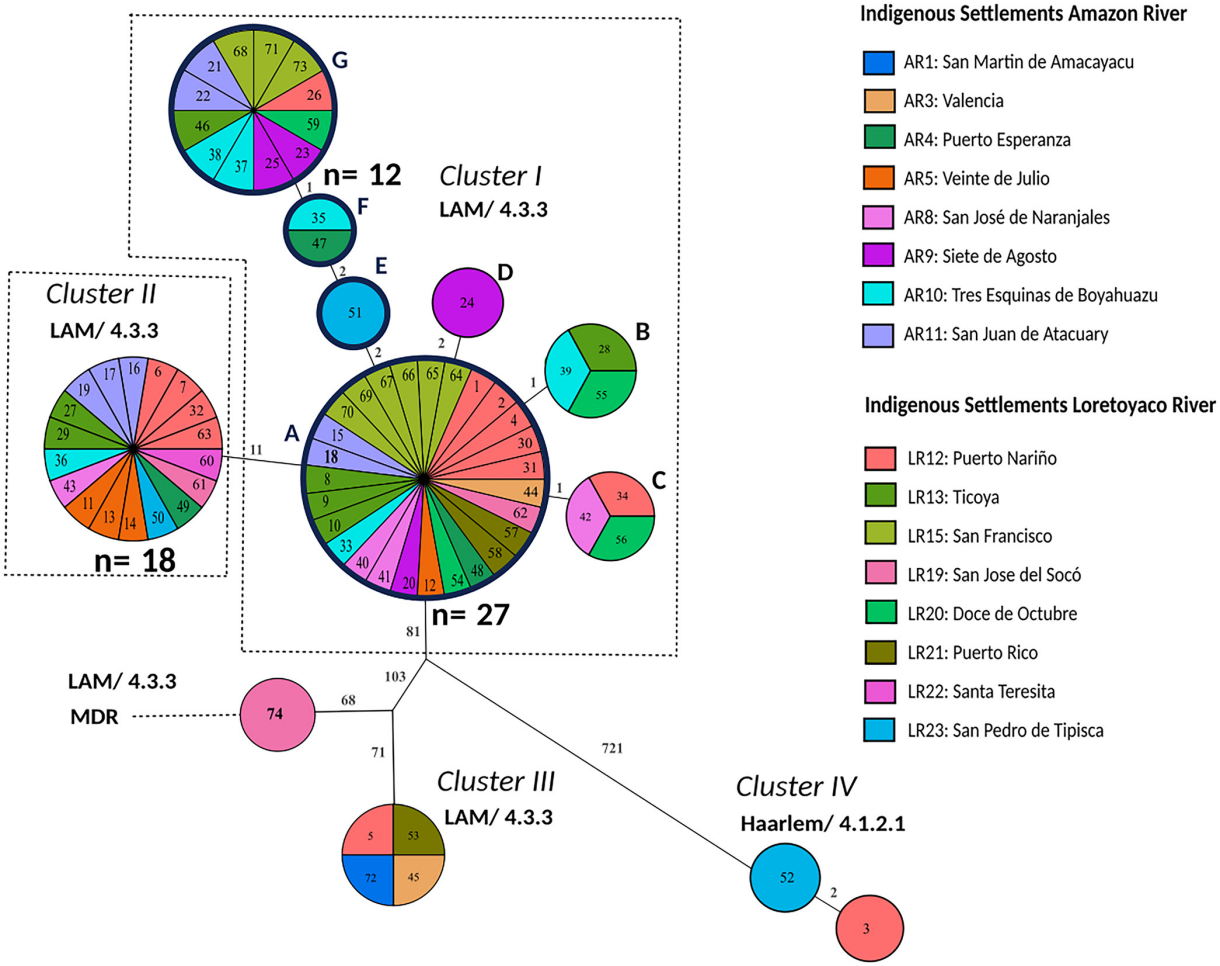
Genetic relationship of patient isolates based on an H37Rv reference mapping approach, including repetitive regions and drug resistance-associated genes. The maximum parsimony tree (MPT) was based on 1,062 single nucleotide polymorphisms (SNPs) of 74 Mycobacterium tuberculosis complex (MTBC) isolates recovered from 16 indigenous settlements. The size of each node is proportional to the number of isolates. The genetic distance is indicated on branches as the number of SNPs that differ between nodes. Samples are color-coded based on the patient’s settlement and identified with an ID number. In the data sets, the sample ID number is preceded by the label COL, which refers to Colombia. Outbreak clusters are named I, II, III, and IV. Clusters comprising the clonal events are shown with dashed boxes, and nodes of cluster I are termed A, B, C, and D. New nodes derived from node A are termed E, F, and G. LAM, Latin American Mediterranean; MDR, multidrug-resistant; AR, Amazon River; LR, Loretoyaco River.

In a first attempt to increase genotyping resolution, we explored whether the inclusion of SNPs in repetitive and resistance-associated genes increases the typing resolution for the outbreak. This approach improved the phylogenetic resolution and led to a subdivision of the largest cluster, cluster I (Fig. 4). Overall, the number of SNPs included in the concatenated SNP alignment rose from 890 to 1,062 SNPs, resulting in the increase in the number of nodes in the outbreak from 5 to 8 and 3 additional branches in the MPT, i.e., three new putative transmission links (Table 2; Fig. 4). Cluster I was split into three new nodes: one isolate at a 2-SNP distance (node E), two isolates at a 4-SNP distance (node F), and 12 isolates (node D) at a 5-SNP distance toward the parental node A (Fig. 4). The 12 isolates within node G were characterized by the presence of 5 novel SNPs (Table 3): *Rv1194c* Trp244Arg, *Rv3621c* (*PPE65*) Gln253Arg, *Rv3478* (*PPE60*), *Rv3478* (*PPE60*) Ala326Ala, and *Rv1945* Ala327Ala.

**TABLE 2:** Details of the reference mapping approach^*a*^

Mapping approach	Standard	Including RR and DRAG	New outbreak genome^*b*^
Reference genome	H37Rv	H37Rv	LAM/4.3.3 (COL-2)
GenBank accession no.	NC_000962.3	NC_000962.3	ERR10558090
Reference genome size (bp)	4,411,532	4,411,532	4,402,676
GC content (×)	65.61	65.61	65.61
No. of CDS	4,069	4,069	4,067
No. of differentiating SNPs	890	1,062	1,094
No. outbreak nodes in the MPT	5	8	9
No. of branches differentiating the outbreak	4	7	9

a LAM, Latin American Mediterranean; SNPs, single nucleotide polymorphisms; bp, bases pairs; GC, guanine cytosine; MPT, maximum parsimony tree; RR, repetitive regions; DRAG, drug resistance-associated genes; CDS, coding sequences based on Prokka annotation.

b Including repetitive regions and resistance genes.

**TABLE 3:** Description of 16 mutations differentiating the clonal clusters of the LAM/4.3.3 outbreak

SNP no.	Genomic position	R^*a*^	A^*b*^	Locus	Gene name	Annotation	Amino acid change (codon change)	LAM^*c*^ cluster
1	1337784	A	G	*Rv1194c*		Hypothetical protein	W244R (tgg/Cgg)	I
2	4061132	T	C	*Rv3621c*	*PPE65*	PPE family protein PPE65	Q253R (cag/cGg)	I
3	3895400	A	G	*Rv3478*	*PPE60*	PE family protein PPE60	P325P (cca/ccG)	I
4	3895403	A	C	*Rv3478*	*PPE60*	PE family protein PPE60	A326A (gca/gcC)	I
5	2196969	G	C	*Rv1945*	-	Hypothetical protein	A327A (gcg/gcC)	I
6	78383	T	C	*Rv0070c*	*glyA2*	Serine hydroxymethyltransferase GlyA2 (serine methylase 2) (SHMT 2)	I172V (atc/Gtc)	II
7	413981	T	C	*Rv0343*	*iniC*	Isoniazid inducible gene protein IniC	S409P (tcg/Ccg)	II
8	637055	C	T	*Rv0545c*	*pitA*	Probable low-affinity inorganic phosphate transporter integral membrane protein PitA	V137M (gtg/Atg)	II
9	758066	G	C	*Rv0663*	*atsD*	Possible arylsulfatase AtsD (aryl-sulfate sulphohydrolase) (arylsulphatase)	G644R (ggc/Cgc)	II
10	960798	C	G	*Rv0862c*		Hypothetical protein	A605A (gcg/gcC)	II
11	1062381	C	T	*Rv0951*	*sucC*	Probable succinyl-CoA synthetase (beta chain) SucC (SCS-beta)	P140S (ccg/Tcg)	II
12	1158574	C	T	*Rv1033c*	*trcR*	Two components of transcriptional regulator TrcR	G55S (ggc/Agc)	II
13	1290447	G	C	*Rv1161*	*narG*	Respiratory nitrate reductase (alpha chain) NarG	M1040I (atg/atC)	II
14	1403781	C	T	*Rv1256c*	*cyp130*	Probable cytochrome P450 130 Cyp130	G275S (ggc/Agc)	II
15	2229203	C	T	*Rv1985c*		Probable transcriptional regulatory protein (probably LysR-family)	G234R (ggg/Agg)	II
16	3461997	G	C	*Rv3093c*	-	Hypothetical oxidoreductase	S256S (tcc/tcG)	II

a R, reference allele.

b A, alternative allele.

c LAM, Latin American Mediterranean.

**FIG 5:**
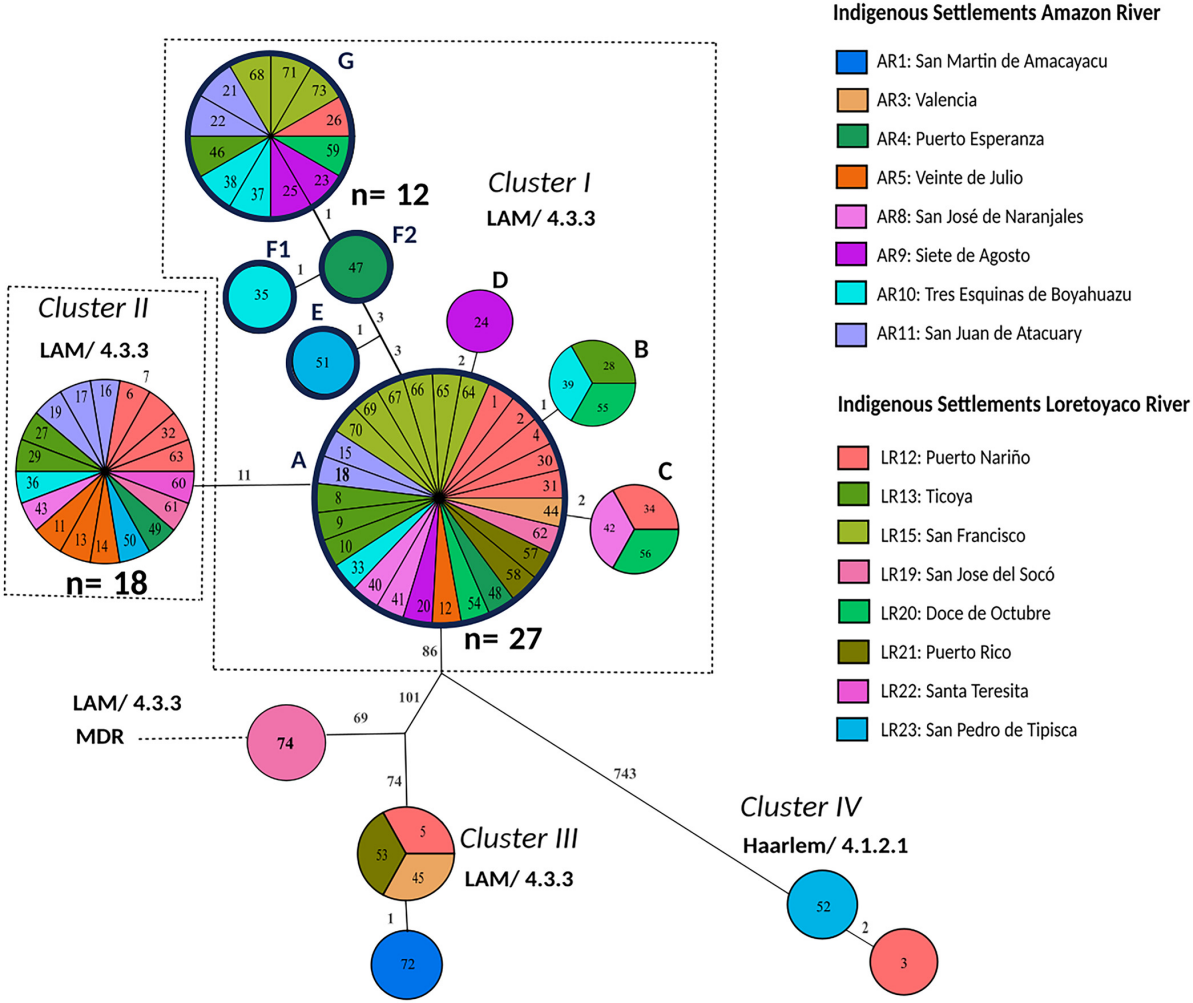
Genetic relationships of patient isolates based on a mapping approach including repetitive regions, drug resistance-associated genes, and a newly assembled genome from the outbreak strain 2 as reference. The MPT is based on 1,094 SNPs of 74 Mycobacterium tuberculosis complex (MTBC) isolates from 16 indigenous settlements. The size of each node is proportional to the number of isolates. The genetic distance is indicated on branches as the number of SNPs that differ between nodes. Samples are color-coded based on the patient’s settlement and identified with an ID number. In the data sets, the sample ID number is preceded by the label COL, which refers to Colombia. Outbreak clusters are named I, II, III, and IV. Clusters comprising the clonal events are shown with dashed boxes, and their nodes are termed A, B, C, and D. New nodes derived from node A are termed E, F, and G. The partition of node F into F1 and F2 is shown. LAM, Latin American Mediterranean; MDR, multidrug-resistant; AR, Amazon River; LR, Loretoyaco River.

Next, we assembled the outbreak-specific genome from isolate COL-2 *de novo* and used it as a reference genome. The COL-2 genome comprised 4,402,676 nucleotides (two contigs, 3,947,682 and 454,994 bp) and was slightly smaller than the H37Rv reference genome with 4,411,532 nucleotides (Table 2). The gene content between COL-2 and H37Rv was highly similar; however, we observed 6 disrupted and 10 newly annotated coding sequences due to larger insertions and deletions (Table 2 and Table S2). Employing the COL-2 genome as a reference for the mapping approach resulted in a further increase in the number of differentiating SNPs in the concatenated sequence from 1,062 to 1,094, which also resulted in an increase of outbreak nodes in the MPT from 8 to 9 (Table 2). The overall population structure in the MPT remained consistent, and node F was further differentiated into F1 and F2, resulting in two new branches in the MPT (Fig. 5).

**FIG 6:**
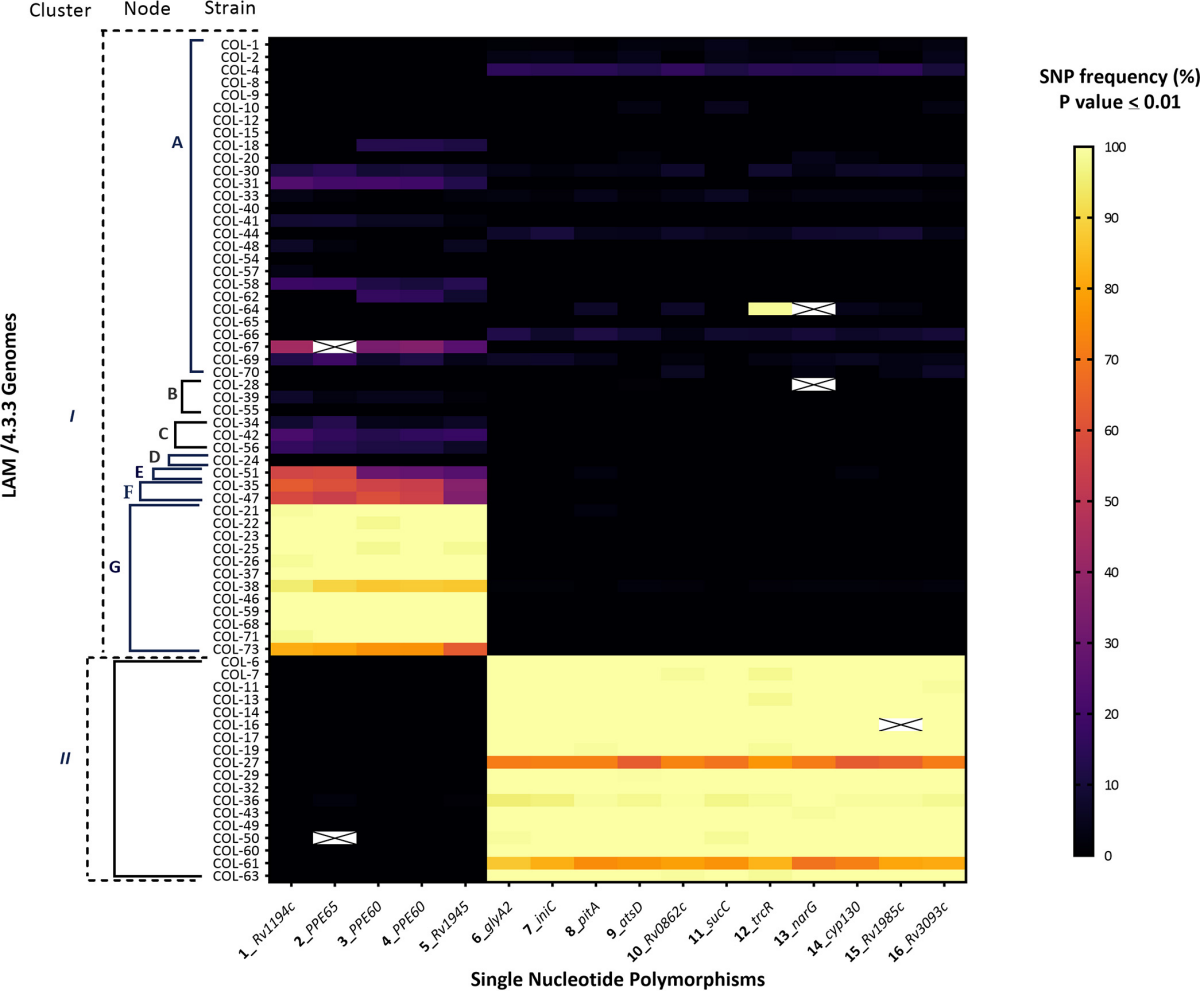
Heat map representing the intrapatient diversity of 16 outbreak-differentiating variants. The *y* axis shows 67 LAM/4.3.3 genomes composing the clonal outbreak; their corresponding nodes and clusters are indicated with solid and dashed brackets, respectively. The *x* axis shows 16 genes comprising SNP-differentiating nodes E, F, and G in cluster I (5 SNPs) and cluster II (11 SNPs) (see Fig. 4). SNP frequency is color-coded from a continuous dark violet to yellow. SNPs were called in confidence positions with a *P* value of ≤0.01 considering an H37Rv reference mapping. Empty crossed cells represent individual SNPs that could not be identified in the Illumina short-read data due to a possible deletion (gaps in the alignment of the respective genome positions). SNP, single nucleotide polymorphism; LAM, Latin American Mediterranean; MDR, multidrug-resistant; AR, Amazon River; LR, Loretoyaco River.

Lastly, we investigated potential mixed infections in all outbreak isolates (*n* = 67) based on the allele frequencies of 16 SNPs, differentiating especially cluster I, node G (5 SNPs), and cluster II (11 SNPs) isolates (Fig. 6). Overall, 22/67 isolates were considered clonal infections; i.e., we did not find heterogeneous alleles in any of the 16 genome positions (Table S3). However, in 20/67 isolates, either all cluster II-specific SNPs or all cluster I (node G)-specific SNPs were identified at a lower frequency (<100%), suggesting a mixed infection with at least two clones of the outbreak. In the remaining 25/67 isolates, heterogeneous alleles were present in some but not all of the node-specific positions.

## DISCUSSION

Here, we employed a newly assembled outbreak reference genome and alternative variant calling parameters to enhance the resolution for analysis of an MTBC outbreak compared to classical comparative MTBC genomic analysis. Our alternative high-resolution approach revealed additional putative transmission events, which can improve epidemiological investigations and interventions in MTBC outbreaks or in settings with a very clonal MTBC population structure. Such investigations are urgently needed since indigenous peoples in the Amazon are facing the highest TB burden in the region of the Americas and Colombia but the lack of access to these patients limits our understanding of MTBC population structure and transmission dynamics in these remote settings (2, 23–26). Including repetitive and resistance-associated regions of the reference genome allowed us to detect additional variants differentiating the outbreak and further suggested that multiple patients in this remote setting were likely infected with more than one clone.

In this study, WGS analysis confirmed the clonal expansion of one lineage 4 MTBC strain that infected most of the patients in this remote Amazon setting in our short sampling time from March to October 2016. It has been suggested that L4 strains were introduced into the indigenous pre-Columbian societies, presumably by the European colonization (27). In particular, our finding that LAM 4.3.3 MTBC strains accounted for 97% of the isolates in our study is in line with previous studies reporting LAM MTBC strains as the most common sublineage in northern South America (Ecuador, Peru, Brazil, and Venezuela), followed by Haarlem, Orphan, and T (20).

Our SNP-based phylogenetic analysis revealed that the LAM 4.3.3 outbreak comprises two clusters defined by distinct mutations. Patients within these clusters originate from different settlements, and there was no apparent infection hot spot. However, we speculate that many infections may have occurred in the municipality of Puerto Nariño, which is a major hub in the region and is frequently visited by people from different settlements. In fact, 24 out of 42 cases of the main transmission chain (cluster I, node A) were from Puerto Nariño and nearby settlements such as San Francisco, Ticoya, Veinte de Julio, and Puerto Esperanza, which is the main gathering area (i.e., fishing, shopping, and schooling) and is located the Amazon River port that connects the indigenous settlements with the modern world.

The classical MTBC genomic analysis indeed did not provide a significantly higher resolution than the 24-loci MIRU-VNTR genotyping investigation in our study setting. Here, only an extended variant calling approach could further increase the resolution of this outbreak scenario and identify new yet unrecognized transmission chains. First, we included repetitive regions such as proline-glutamic acid/proline-proline-glutamic-acid (PE/PPE) and resistance-associated genes in our analysis. Of note, PPE/PE genes account for nearly 10% of the genes of MTBC and might play a role in the virulence mechanisms of host-pathogen interaction (28, 29); however, their high guanine cytosine (GC) content and sequence repetitions challenge standard WGS pipelines (22, 30, 31). Nevertheless, recent work has shown that the inclusion of PPE/PE genes with strict variant calling thresholds does not negatively impact the analysis and can provide novel and reliable information on mutations (31, 32).

Second, we assembled a new reference genome from an outbreak isolate of the largest node, node A, using long-read sequencing data. Molecular epidemiological approaches in recent years mostly have used H37Rv as a mapping reference. However, its use as a sole reference genome has been discussed, as this does not consider the genetic diversity across all 9 distinct human-adapted MTBC lineages and sublineages (33, 34). Thus, it is intriguing to consider whether the use of local reference genomes enables an improved resolution power, especially those caused by lineages other than lineage 4. Along these lines, Lee et al. have suggested deep sequencing in combination with the assembly of an outbreak-specific reference genome based on long-read sequencing as an alternative approach to enhance resolution and identify superspreaders in highly clonal outbreaks (35). However, this method is limited by high costs. Here, both approaches, i.e., including repetitive regions for variant calling and a new outbreak reference genome, led to an increased outbreak resolution and a number of differentiating SNPs.

With this new resolution, we also found evidence that multiple patients are potentially infected with more than one outbreak strain. Others, however, have found considerable intrapatient heterogeneity, often related to one infecting clone (36). Previous work has documented that mixed infections might account for 30% of TB cases, for which the mixed infection rates, i.e., with distinct strains, are estimated to be from 19% in pulmonary samples to 51% in extrapulmonary and pulmonary combined samples (37–40). However, in settings with a clonal population structure and with classical comparative MTBC genomic pipelines, the assessment of mixed infections is extremely difficult (41). This is also particularly true for regions with a high incidence of MDR-TB, which are often dominated by few major clades with drastically reduced genetic diversity (42, 43). Here, it is crucial for molecular diagnostics to also identify minority MTBC populations in one patient, which could have a huge impact on diagnosis, treatment decisions, and clinical outcomes.

Our study has limitations, such as the short study duration, rendering the comprehensive analysis of the TB epidemiology in the study region difficult. Future work should be based on longitudinal sampling enabling prospective molecular surveillance and allowing us to trace the evolution of drug resistance in locally dominating clades/strains. Our analysis was focused on SNPs, and the inclusion of insertions and deletions could further improve the phylogenetic resolution.

**Conclusions.** In conclusion, we confirmed that the TB epidemiology in the indigenous setting of Puerto Nariño in the Southwest Colombian Amazon is mainly driven by the ongoing transmission of one outbreak strain. Almost one-third of the patients were possibly infected with at least two clones of the local outbreak strain, highlighting the need for interventions to break transmission chains and better TB control in the population. We also demonstrated that customized variant calling pipelines and a new local reference genome can moderately increase the resolution of MTBC outbreaks. The potential benefit of these customized reference-mapping approaches for molecular surveillance studies needs to be investigated in other outbreaks and may vary depending on the strain genetic background.

## MATERIALS AND METHODS

### Patient cohort.

We recovered a total of 74 MTBC isolates from primary sputum samples on Löwenstein-Jensen medium (LJ) from March to October 2016. These samples were obtained from patients with pulmonary TB allocated to 16 indigenous settlements based on the shore of the Amazon (AR) and Loretoyaco Rivers (LR) in Puerto Nariño (19). The sociodemographic and clinical characteristics of the TB patients were recorded. These included Age, sex, health system affiliation, household conditions, history of TB, Bacillus Calmette-Guérin (BCG) vaccination, recreational drug usage, alcohol, smoking, self-reported human immunodeficiency virus (HIV) status, and other medical diagnoses.

### Statistical analysis.

We calculated descriptive statistics of the demographic data of the population studied, including proportions and median and interquartile ranges using the functions of base R and RStudio (43).

### DNA extraction and classical genotyping.

We extracted genomic DNA using the PureLink genomic DNA minikit (Thermo Fisher Scientific) according to the manufacturer’s instructions. DNA samples were genotyped using 24-loci mycobacterial interspersed repetitive-unit–variable-number tandem-repeat (MIRU-VNTR) as follows. Multiplex PCR amplification was performed with a GenoScreen typing kit, and fragment separation was run on the ABI 3500XL automated sequencer as described previously (45). Fragment analysis was accomplished using GeneMapper software (PE Applied Biosystems). Genotypes were identified on the web-based databank MIRU-VNTR*plus* (https://www.miru-vntrplus.org/MIRU/index.faces). Phylogenetic analyses were performed with BioNumerics software version 7.6 (Applied Maths). MIRU clusters comprised nodes with more than one isolate with an identical genotype. A 24-loci MIRU-VNTR dendrogram was calculated using the categorical distance and the unweighted pair group method with an arithmetic mean algorithm (UPGMA).

### Whole-genome sequencing and bioinformatic analysis.

For WGS, DNA libraries were prepared based on the Baym protocol with the Nextera XT kits and sequenced with the NextSeq 500 sequencing platform from Illumina (151 bp, paired end) according to the manufacturer’s instructions (46). We analyzed the sequencing data with the MTBseq pipeline, thus using a reference mapping approach (22, 42, 47) and inferring the phylogenetic lineage and resistance-associated variants (48). Briefly, FASTQ files were mapped to the reference genome Mycobacterium tuberculosis H37Rv (GenBank ID NC_000962.3) using the Burrows-Wheeler Aligner Alignment tool (49), and refined mapping was done with the Genome Analysis Toolkit software package (50). For inference of the phylogeny, a concatenated SNP alignment was built from SNP positions with the MTBseq default thresholds: 4 reads mapped in forward and reverse orientations, a minimum of 4 reads supporting the allele with a Phred score of not less than 20, and 75% allele frequency. We excluded SNP positions within repetitive regions and drug resistance-associated genes and finally combined positions that matched these thresholds in >95% of the isolates. We submitted sequencing data to the European Nucleotide Archive (accession numbers are given in Table S1). In addition, we generated a concatenated SNP alignment with the thresholds mentioned above but also including repetitive regions (51).

Cluster analysis was performed using the MTBseq pipeline with a threshold of 5 SNPs (48, 52).

A new reference genome of one outbreak strain named COL-2 was assembled using the Single Molecule Real Time (SMRT), long-read technology of the Sequel II system (Pacific Biosciences [PacBio]) to increase the resolution of the outbreak. We selected the local isolate COL-2 from the largest clonal cluster to serve as our reference. The DNA library was prepared with the SMRTbell Express Template prep kit version 2.0 with barcoded adapters from Integrated DNA Technologies (IDT) and sequenced on the Sequel II system. *De novo* genome assembly was performed using the PacBio SMRTlink software version 9.0 and its microbial assembly application, with the genome length set to 4.4 Mb and a seed coverage of 30. The assembly of 54,267 subreads (*N*_50_ length, 6,696 bp; coverage, 73.5×) resulted in two polished contiguous sequences (contigs) of 3,947,682 and 454,994 bp, respectively.

Maximum parsimony trees (MPT) were generated with BioNumerics either based on a distance matrix derived from the standard 24-loci MIRU-VNTR data or derived from the three reference-mapping approaches based on a concatenated SNP alignment with classical SNP calling thresholds: (i) the classical comparative MTBC genomic pipeline, (ii) including repetitive regions and drug resistance-associated regions, and (iii) reference mapping to a new outbreak genome and including repetitive regions. All approaches employed the default MTBseq conservative SNP-calling parameters described above. In the data sets, the sample ID number is preceded by the label COL- which refers to Colombia; however, only the ID number is depicted on the MPT.

We searched the binary alignment map (BAM) files of the outbreak isolates (*n* = 67) for variants with variable allele frequencies in 16 outbreak-differentiating SNP positions using binoSNP as well as in resistance-associated regions (i.e., alternative alleles with *P* values of <0.01 were considered) (53). The 16 phylogenetic informative positions were further validated with a visual inspection of the bam.gatk alignment files using the integrative genome viewer (IGV). The frequencies of these variants were represented for all outbreak isolates in a heat map using GraphPad Prism version 9 software.

To identify the corresponding positions of variants of interest in the PacBio assembly, we performed a genome alignment between H37Rv (lineage 4 [L4]) (GenBank ID NC_000962.3) (54–56) and PacBio COL-2 (L4, Latin American Mediterranean [LAM]) reference genomes using the Mauve plugin (57) with Geneious Prime software. Both genomes were annotated with Prokka with default parameters to identify new genes and putative loss/gain of functions due to larger insertions and deletions (58). Repetitive regions, mobile elements, hypothetical genes, and smaller insertions and deletions not affecting the coding sequence annotation are not listed in Table S2.

The geographical maps shown are an adaptation of Google and Colombian National Maps: Comunidades Indígenas de Puerto Nariño and Asociación de Indígenas, Tikuna, Cocama and Yagua (ATICOYA). Open data were provided by Gobernación del Amazonas at https://www.datos.gov.co.

### Data availability.

The Illumina short-read sequences of the 74 genomes of the outbreak and the PacBio long-read sequence corresponding to the local reference genome COL-2 are publicly accessible in the European Nucleotide Archive under the projects PRJEB57971 and PRJEB57950 (see Table S1).

## References

[1] World Health Organization. 2020. Global tuberculosis report 2020. World Health Organization, Geneva, Switzerland.

[2] Pan American Health Organization. 2018. Tuberculosis in the Americas, 2018. PAHO, Washington, DC.

[3] World Health Organization. 2021. Global tuberculosis report 2021. World Health Organization, Geneva, Switzerland.

[4] Instituto Nacional de Salud. 2020. Comportamiento de la vigilancia de tuberculosis, Colombia, 2020. Instituto Nacional de Salud, Bogota, Colombia. https://www.ins.gov.co/buscador-eventos/BoletinEpidemiologico/2021_Boletin_epidemiologico_semana_11.pdf.

[5] Instituto Nacional de Salud. 2016. Informe Evento Tuberculosis. Instituto Nacional de Salud, Bogota, Colombia. http://www.ins.gov.co/buscador-eventos/Informesdeevento/Tuberculosis%20%202016.pdf.

[6] Pan American Health Organization. 2019. Tuberculosis in the Americas. 2019 regional report. Pan American Health Organization/Editorial El Manual Moderno, Washington, DC./ Mexico City, Mexico.

[7] World Health Organization. 2019. 2019 antibacterial agents in clinical development: an analysis of the antibacterial clinical development pipeline. World Health Organization, Geneva, Switzerland.

[8] World Bank Group. 2015. Indigenous Latin America in the twenty-first century: the first decade. World Bank Group, Washington, DC. https://openknowledge.worldbank.org/entities/publication/1b122e3f-7058-5f6c-bf7d-7d88873cc5ad.

[9] Cormier M, Schwartzman K, N’Diaye DS, Boone CE, Dos Santos AM, Gaspar J, Cazabon D, Ghiasi M, Kahn R, Uppal A, Morris M, Oxlade O. 2019. Proximate determinants of tuberculosis in Indigenous peoples worldwide: a systematic review. Lancet Glob Health 7:e68–e80. doi:10.1016/S2214-109X(18)30435-2. 30554764

[10] Ramírez JD, Sordillo EM, Gotuzzo E, Zavaleta C, Caplivski D, Navarro JC, Crainey JL, Bessa Luz SL, Noguera LAD, Schaub R, Rousseau C, Herrera G, Oliveira-Miranda MA, Quispe-Vargas MT, Hotez PJ, Mondolfi AP. 2020. SARS-CoV-2 in the Amazon region: a harbinger of doom for Amerindians. PLoS Negl Trop Dis 14:e0008686. doi:10.1371/journal.pntd.0008686. 33119616 PMC7595282

[11] United Nations. 2020. Report of the Secretary-General. Progress towards achieving global tuberculosis targets and implementation of the UN political declaration on tuberculosis. Agenda item 132. Global health and foreign policy. https://digitallibrary.un.org/record/3887628.

[12] World Health Organization. 2018. The use of next-generation sequencing technologies for the detection of mutations associated with drug resistance in Mycobacterium tuberculosis complex: technical guide. WHO, Geneva, Switzerland.

[13] United Nations. 2018. Political declaration of the UN General-Assembly High-Level Meeting on the Fight Against Tuberculosis. United Nations, New York, NY.

[14] Meehan CJ, Goig GA, Kohl TA, Verboven L, Dippenaar A, Ezewudo M, Farhat MR, Guthrie JL, Laukens K, Miotto P, Ofori-Anyinam B, Dreyer V, Supply P, Suresh A, Utpatel C, van Soolingen D, Zhou Y, Ashton PM, Brites D, Cabibbe AM, de Jong BC, de Vos M, Menardo F, Gagneux S, Gao Q, Heupink TH, Liu Q, Loiseau C, Rigouts L, Rodwell TC, Tagliani E, Walker TM, Warren RM, Zhao Y, Zignol M, Schito M, Gardy J, Cirillo DM, Niemann S, Comas I, Van Rie A. 2019. Whole genome sequencing of Mycobacterium tuberculosis: current standards and open issues. Nat Rev Microbiol 17:533–545. doi:10.1038/s41579-019-0214-5. 31209399

[15] Vargas R, Freschi L, Marin M, Epperson LE, Smith M, Oussenko I, Durbin D, Strong M, Salfinger M, Farhat MR. 2021. In-host population dynamics of Mycobacterium tuberculosis complex during active disease. Elife 10:e61805. doi:10.7554/eLife.61805. 33522489 PMC7884073

[16] Nimmo C, Brien K, Millard J, Grant AD, Padayatchi N, Pym AS, O’Donnell M, Goldstein R, Breuer J, Balloux F. 2020. Dynamics of within-host Mycobacterium tuberculosis diversity and heteroresistance during treatment. EBioMedicine 55:102747. doi:10.1016/j.ebiom.2020.102747. 32361247 PMC7195533

[17] England World Leaders in the Use of Whole Genome Sequencing to Diagnose TB. 2017. Whole genome sequencing (WGS) is now being used to identify different strains of tuberculosis (TB), announced Public Health England today. https://www.gov.uk/government/news/england-world-leaders-in-the-use-of-whole-genome-sequencing-to-diagnose-tb. Retrieved 25 July 2021.

[18] Shea J, Halse TA, Lapierre P, Shudt M, Kohlerschmidt D, Van Roey P, Limberger R, Taylor J, Escuyer V, Musser KA. 2017. Comprehensive whole-genome sequencing and reporting of drug resistance profiles on clinical cases of Mycobacterium tuberculosis in New York State. J Clin Microbiol 55:1871–1882. doi:10.1128/JCM.00298-17. 28381603 PMC5442544

[19] Marín AV, Rastogi N, Couvin D, Mape V, Murcia MI. 2021. First approach to the population structure of Mycobacterium tuberculosis complex in the indigenous population in Puerto Nariño-Amazonas, Colombia. PLoS One 16:e0245084. doi:10.1371/journal.pone.0245084. 33411781 PMC7790298

[20] García C. 2019. Situación de la Tuberculosis Pulmonar en Población Indígena con Asentamiento en Puerto Nariño – Amazonas, Año 2016. Universidad Nacional de Colombia, Bogota, Colombia.

[21] Cerezo-Cortés M, Rodríguez-Castillo J, Hernández-Pando R, Murcia M. 2019. Circulation of M. tuberculosis Beijing genotype in Latin America and the Caribbean. Pathog Glob Health 113:336–351. doi:10.1080/20477724.2019.1710066. 31903874 PMC7006823

[22] Coll F, McNerney R, Guerra-Assunção JA, Glynn JR, Perdigão J, Viveiros M, Portugal I, Pain A, Martin N, Clark TG. 2014. A robust SNP barcode for typing Mycobacterium tuberculosis complex strains. Nat Commun 5:4812. doi:10.1038/ncomms5812. 25176035 PMC4166679

[23] Ranzani OT, Pescarini JM, Martinez L, Garcia-Basteiro AL. 2021. Increasing tuberculosis burden in Latin America: an alarming trend for global control efforts. BMJ Glob Health 6:e005639. doi:10.1136/bmjgh-2021-005639. PMC7993346 33762254

[24] Kolte IV, Pereira L, Benites A, de Sousa IMC, Basta PC. 2020. The contribution of stigma to the transmission and treatment of tuberculosis in a hyperendemic indigenous population in Brazil. PLoS One 15:e0243988. doi:10.1371/journal.pone.0243988. 33326453 PMC7743939

[25] Polanco-Pasaje JE, Rodríguez-Márquez I, Tello-Hoyos KY, Torres-Pereda P, Guzmán-Salazar BL, Pérez F. 2021. Tuberculosis care cascade for the indigenous population in Colombia: an operational research study. Rev Panam Salud Publica 45:e20. doi:10.26633/RPSP.2021.20. 33643402 PMC7901045

[26] Tollefson D, Bloss E, Fanning A, Redd JT, Barker K, McCray E. 2013. Burden of tuberculosis in indigenous peoples globally: a systematic review. Int J Tuber Lung Dis 17:1139–1150. doi:10.5588/ijtld.12.0385. PMC6057791 23823137

[27] Brynildsrud OB, Pepperell CS, Suffys P, Grandjean L, Monteserin J, Debech N, Bohlin J, Alfsnes K, Pettersson JO-H, Kirkeleite I, Fandinho F, da Silva MA, Perdigao J, Portugal I, Viveiros M, Clark T, Caws M, Dunstan S, Thai PVK, Lopez B, Ritacco V, Kitchen A, Brown TS, van Soolingen D, O'Neill MB, Holt KE, Feil EJ, Mathema B, Balloux F, Eldholm V. 2018. Global expansion of Mycobacterium tuberculosis lineage 4 shaped by colonial migration and local adaptation. Sci Adv 4:eaat5869. doi:10.1126/sciadv.aat5869. 30345355 PMC6192687

[28] Fishbein S, van Wyk N, Warren RM, Sampson SL. 2015. Phylogeny to function: PE/PPE protein evolution and impact on Mycobacterium tuberculosis pathogenicity. Mol Microbiol 96:901–916. doi:10.1111/mmi.12981. 25727695

[29] Ates LS. 2020. New insights into the mycobacterial PE and PPE proteins provide a framework for future research. Mol Microbiol 113:4–21. doi:10.1111/mmi.14409. 31661176 PMC7028111

[30] Ezewudo M, Borens A, Chiner-Oms Á, Miotto P, Chindelevitch L, Starks AM, Hanna D, Liwski R, Zignol M, Gilpin C, Niemann S, Kohl TA, Warren RM, Crook D, Gagneux S, Hoffner S, Rodrigues C, Comas I, Engelthaler DM, Alland D, Rigouts L, Lange C, Dheda K, Hasan R, McNerney R, Cirillo DM, Schito M, Rodwell TC, Posey J. 2018. Integrating standardized whole genome sequence analysis with a global Mycobacterium tuberculosis antibiotic resistance knowledgebase. Sci Rep 8:15382. doi:10.1038/s41598-018-33731-1. 30337678 PMC6194142

[31] Phelan JE, Coll F, Bergval I, Anthony RM, Warren R, Sampson SL, Gey van Pittius NC, Glynn JR, Crampin AC, Alves A, Bessa TB, Campino S, Dheda K, Grandjean L, Hasan R, Hasan Z, Miranda A, Moore D, Panaiotov S, Perdigao J, Portugal I, Sheen P, de Oliveira Sousa E, Streicher EM, van Helden PD, Viveiros M, Hibberd ML, Pain A, McNerney R, Clark TG. 2016. Recombination in pe/ppe genes contributes to genetic variation in Mycobacterium tuberculosis lineages. BMC Genomics 17:151. doi:10.1186/s12864-016-2467-y. 26923687 PMC4770551

[32] Walter KS, Colijn C, Cohen T, Mathema B, Liu Q, Bowers J, Engelthaler DM, Narechania A, Lemmer D, Croda J, Andrews JR. 2020. Genomic variant-identification methods may alter Mycobacterium tuberculosis transmission inferences. Microb Genom 6:mgen000418. doi:10.1099/mgen.0.000418. 32735210 PMC7641424

[33] O’Toole RF, Gautam SS. 2017. Limitations of the Mycobacterium tuberculosis reference genome H37Rv in the detection of virulence-related loci. Genomics 109:471–474. doi:10.1016/j.ygeno.2017.07.004. 28743540

[34] Bush SJ, Foster D, Eyre DW, Clark EL, De Maio N, Shaw LP, Stoesser N, Peto TEA, Crook DW, Walker AS. 2020. Genomic diversity affects the accuracy of bacterial single-nucleotide polymorphism-calling pipelines. Gigascience 9:giaa007. doi:10.1093/gigascience/giaa007. 32025702 PMC7002876

[35] Lee RS, Proulx J-F, McIntosh F, Behr MA, Hanage WP. 2020. Previously undetected super-spreading of *Mycobacterium tuberculosis* revealed by deep sequencing. eLife 9:e53245. 32014110 10.7554/eLife.53245 PMC7012596

[36] Merker M, Kohl TA, Roetzer A, Truebe L, Richter E, Rüsch-Gerdes S, Fattorini L, Oggioni MR, Cox H, Varaine F, Niemann S. 2013. Whole genome sequencing reveals complex evolution patterns of multidrug-resistant Mycobacterium tuberculosis Beijing strains in patients. PLoS One 8:e82551. doi:10.1371/journal.pone.0082551. 24324807 PMC3855793

[37] McIvor A, Koornhof H, Kana BD. 2017. Relapse, re-infection and mixed infections in tuberculosis disease. Pathog Dis 75:ftx020. doi:10.1093/femspd/ftx020. 28334088

[38] Cohen T, van Helden PD, Wilson D, Colijn C, McLaughlin MM, Abubakar I, Warren RM. 2012. Mixed-strain mycobacterium tuberculosis infections and the implications for tuberculosis treatment and control. Clin Microbiol Rev 25:708–719. doi:10.1128/CMR.00021-12. 23034327 PMC3485752

[39] Shamputa IC, Jugheli L, Sadradze N, Willery E, Portaels F, Supply P, Rigouts L. 2006. Mixed infection and clonal representativeness of a single sputum sample in tuberculosis patients from a penitentiary hospital in Georgia. Respir Res 7:99. doi:10.1186/1465-9921-7-99. 16846493 PMC1538999

[40] Shamputa IC, Rigouts L, Eyongeta LA, El Aila NA, van Deun A, Salim AH, Willery E, Locht C, Supply P, Portaels F. 2004. Genotypic and phenotypic heterogeneity among Mycobacterium tuberculosis isolates from pulmonary tuberculosis patients. J Clin Microbiol 42:5528–5536. doi:10.1128/JCM.42.12.5528-5536.2004. 15583277 PMC535260

[41] Galagan JE. 2014. Genomic insights into tuberculosis. Nat Rev Genet 15:307–320. doi:10.1038/nrg3664. 24662221

[42] Merker M, Blin C, Mona S, Duforet-Frebourg N, Lecher S, Willery E, Blum MGB, Rüsch-Gerdes S, Mokrousov I, Aleksic E, Allix-Béguec C, Antierens A, Augustynowicz-Kopeć E, Ballif M, Barletta F, Beck HP, Barry CE, Bonnet M, Borroni E, Campos-Herrero I, Cirillo D, Cox H, Crowe S, Crudu V, Diel R, Drobniewski F, Fauville-Dufaux M, Gagneux S, Ghebremichael S, Hanekom M, Hoffner S, Jiao W, Kalon S, Kohl TA, Kontsevaya I, Lillebæk T, Maeda S, Nikolayevskyy V, Rasmussen M, Rastogi N, Samper S, Sanchez-Padilla E, Savic B, Shamputa IC, Shen A, Sng L-H, Stakenas P, Toit K, Varaine F, Vukovic D, et al. 2015. Evolutionary history and global spread of the Mycobacterium tuberculosis Beijing lineage. Nat Genet 47:242–249. doi:10.1038/ng.3195. 25599400 PMC11044984

[43] Casali N, Nikolayevskyy V, Balabanova Y, Harris SR, Ignatyeva O, Kontsevaya I, Corander J, Bryant J, Parkhill J, Nejentsev S, Horstmann RD, Brown T, Drobniewski F. 2014. Evolution and transmission of drug resistant tuberculosis in a Russian population. Nat Genet 46:279–286. doi:10.1038/ng.2878. 24464101 PMC3939361

[44] R Core Team. 2022. 2022. R: a language and environment for statistical computing. R Foundation for Statistical Computing, Vienna, Austria. https://www.r-project.org/.

[45] Supply P, Allix C, Lesjean S, Cardoso-Oelemann M, Rüsch-Gerdes S, Willery E, Savine E, de Haas P, van Deutekom H, Roring S, Bifani P, Kurepina N, Kreiswirth B, Sola C, Rastogi N, Vatin V, Gutierrez MC, Fauville M, Niemann S, Skuce R, Kremer K, Locht C, van Soolingen D. 2006. Proposal for standardization of optimized mycobacterial interspersed repetitive unit-variable-number tandem repeat typing of Mycobacterium tuberculosis. J Clin Microbiol 44:4498–4510. doi:10.1128/JCM.01392-06. 17005759 PMC1698431

[46] Baym M, Kryazhimskiy S, Lieberman TD, Chung H, Desai MM, Kishony R. 2015. Inexpensive multiplexed library preparation for megabase-sized genomes. PLoS One 10:e0128036. doi:10.1371/journal.pone.0128036. 26000737 PMC4441430

[47] Homolka S, Projahn M, Feuerriegel S, Ubben T, Diel R, Nübel U, Niemann S. 2012. High resolution discrimination of clinical Mycobacterium tuberculosis complex strains based on single nucleotide polymorphisms. PLoS One 7:e39855. doi:10.1371/journal.pone.0039855. 22768315 PMC3388094

[48] Kohl TA, Utpatel C, Schleusener V, De Filippo MR, Beckert P, Cirillo DM, Niemann S. 2018. MTBseq: a comprehensive pipeline for whole genome sequence analysis of Mycobacterium tuberculosis complex isolates. PeerJ 6:e5895. doi:10.7717/peerj.5895. 30479891 PMC6238766

[49] Li H, Durbin R. 2009. Fast and accurate short read alignment with Burrows-Wheeler transform. Bioinformatics 25:1754–1760. doi:10.1093/bioinformatics/btp324. 19451168 PMC2705234

[50] McKenna A, Hanna M, Banks E, Sivachenko A, Cibulskis K, Kernytsky A, Garimella K, Altshuler D, Gabriel S, Daly M, DePristo MA. 2010. The Genome Analysis Toolkit: a MapReduce framework for analyzing next-generation DNA sequencing data. Genome Res 20:1297–1303. doi:10.1101/gr.107524.110. 20644199 PMC2928508

[51] Page AJ, Taylor B, Delaney AJ, Soares J, Seemann T, Keane JA, Harris SRY. 2016. SNP-sites: rapid efficient extraction of SNPs from multi-FASTA alignments. Microb Genom 2:e000056. doi:10.1099/mgen.0.000056. 28348851 PMC5320690

[52] Walker TM, Ip CLC, Harrell RH, Evans JT, Kapatai G, Dedicoat MJ, Eyre DW, Wilson DJ, Hawkey PM, Crook DW, Parkhill J, Harris D, Walker AS, Bowden R, Monk P, Smith EG, Peto TEA. 2013. Whole-genome sequencing to delineate Mycobacterium tuberculosis outbreaks: a retrospective observational study. Lancet Infect Dis 13:137–146. doi:10.1016/S1473-3099(12)70277-3. 23158499 PMC3556524

[53] Dreyer V, Utpatel C, Kohl TA, Barilar I, Gröschel MI, Feuerriegel S, Niemann S. 2020. Detection of low-frequency resistance-mediating SNPs in next-generation sequencing data of Mycobacterium tuberculosis complex strains with binoSNP. Sci Rep 10:7874. doi:10.1038/s41598-020-64708-8. 32398743 PMC7217866

[54] Cole ST, Brosch R, Parkhill J, Garnier T, Churcher C, Harris D, Gordon SV, Eiglmeier K, Gas S, Barry CE, Tekaia F, Badcock K, Basham D, Brown D, Chillingworth T, Connor R, Davies R, Devlin K, Feltwell T, Gentles S, Hamlin N, Holroyd S, Hornsby T, Jagels K, Krogh A, McLean J, Moule S, Murphy L, Oliver K, Osborne J, Quail MA, Rajandream M-A, Rogers J, Rutter S, Seeger K, Skelton J, Squares R, Squares S, Sulston JE, Taylor K, Whitehead S, Barrell BG. 1998. Deciphering the biology of Mycobacterium tuberculosis from the complete genome sequence. Nature 393:537–544. doi:10.1038/31159. 9634230

[55] Camus J-C, Pryor MJ, Médigue C, Cole STY. 2002. Re-annotation of the genome sequence of Mycobacterium tuberculosis H37Rv. Microbiology (Reading) 148:2967–2973. doi:10.1099/00221287-148-10-2967. 12368430

[56] Lew JM, Kapopoulou A, Jones LM, Cole ST. 2011. TubercuList: 10 years after. Tuberculosis (Edinb) 91:1–7. doi:10.1016/j.tube.2010.09.008. 20980199

[57] Darling ACE, Mau B, Blattner FR, Perna NT. 2004. Mauve: multiple alignment of conserved genomic sequence with rearrangements. Genome Res 14:1394–1403. doi:10.1101/gr.2289704. 15231754 PMC442156

[58] Seemann T. 2014. Prokka: rapid prokaryotic genome annotation. Bioinformatics 30:2068–2069. doi:10.1093/bioinformatics/btu153. 24642063

