# Supplementary material for: Transmission Dynamics of a Mycobacterium tuberculosis Complex Outbreak in an Indigenous Population in the Colombian Amazon Region
Source: Microbiol Spectr. 2023 May 24;11(3):e05013-22. doi: 10.1128/spectrum.05013-22 (PMC10269451; doi:10.1128/spectrum.05013-22)
Supplement: Supplemental file 1 — Supplemental material. Download spectrum.05013-22-s0001.pdf, PDF file, 1.3 MB [file spectrum.05013-22-s0001.pdf]

SUPPLEMENTARY MATERIAL

Transmission dynamics of a *Mycobacterium tuberculosis* complex outbreak in an indigenous population in the Colombian Amazon Region

Francy J. Pérez-Llanos [a*], Viola Dreyer [a,b], Ivan Barilar [a,b], Christian Utpatel [a,b], Thomas A. Kohl [a,b], Martha Isabel Murcia[c], Susanne Homolka[a], Matthias Merker [a,b,d,#] & Stefan Niemann [a,b #]

[a]Molecular and Experimental Mycobacteriology, Research Center Borstel, Borstel, Germany

[b]German Center for Infection Research, Hamburg-Lübeck-Borstel-Riems, Germany.

[c]Grupo MICOBAC-UN, Departamento de Microbiología, Facultad de Medicina, Universidad Nacional de Colombia, Bogotá, Colombia

[d]Evolution of the Resistome, Research Center Borstel, Borstel, Germany.

# Address correspondence to Stefan Niemann, sniemann@fz-borstel.de and Matthias Merker, mmerker@fz-borstel.de

*Present address: West German Genome Center, Heinrich-Heine-Universität Düsseldorf, Universitätsklinikum Düsseldorf, Düsseldorf, Germany

Stefan Niemann and Matthias Merker contributed equally to this work. The author’s order was determined based on substantial contribution.

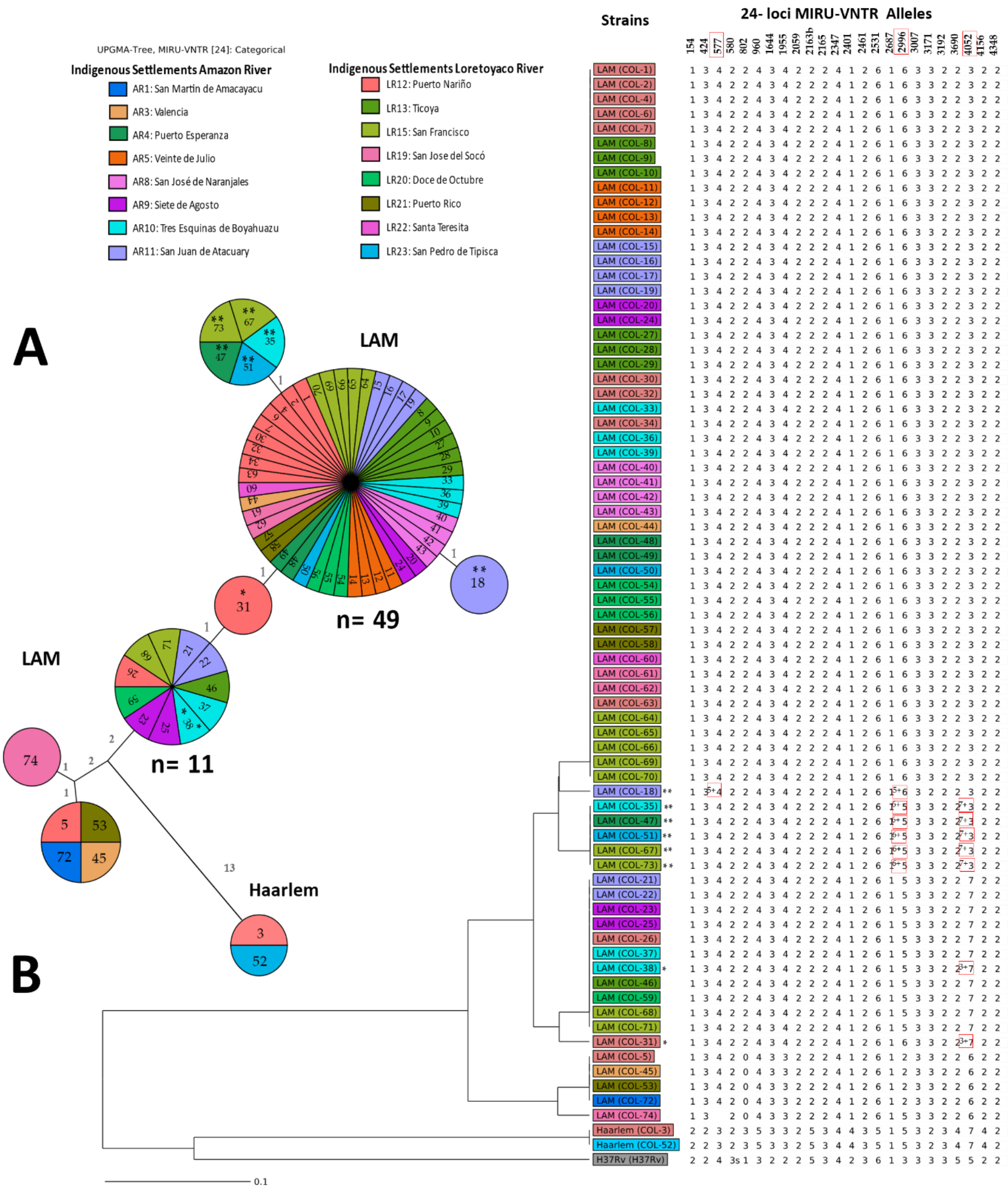

FIG S1 Phylogenetic analysis based on 24-loci MIRU-VNTR data of 74 isolates recovered from the TB outbreak in 16 indigenous settlements in Puerto Nariño during March-October 2016. A) Maximum Parsimony Tree (MPT). Eight nodes are proportional in size to the number of isolates

with identical MIRU-VNTR genotypes. Branches are labeled with the number of MIRU-VNTR alleles that differ between nodes. B) Dendrogram based on the Unweight Pair Group Method with Arithmetic mean algorithm (UPGMA) using www.miru-vntrplus.org. Strains are color-coded based on the indigenous settlement these were recovered. DNA from the H37Rv ATCC reference strain was used as a control. * Samples with one mixed allele: COL-31 (4052, 3+7) and COL -38 (4052, 3+7). ** Samples with two mixed alleles: COL-18 (2996, 5+6) (577, 4+5), COL-35 (2996, 5+6) (4052, 3+7), COL-47 (2996, 5+6) (4052, 3+7), COL-51 (2996, 5+6) (4052, 3+7), COL-67 (2996, 5+6) (4052, 3+7), and COL-73 (2996, 5+6) (4052, 3+7). Red squares indicate the mixed alleles in a given strain. Abbreviations: LAM, Latin American Mediterranean; AR, Amazon River; LR, Loretoyaco River.

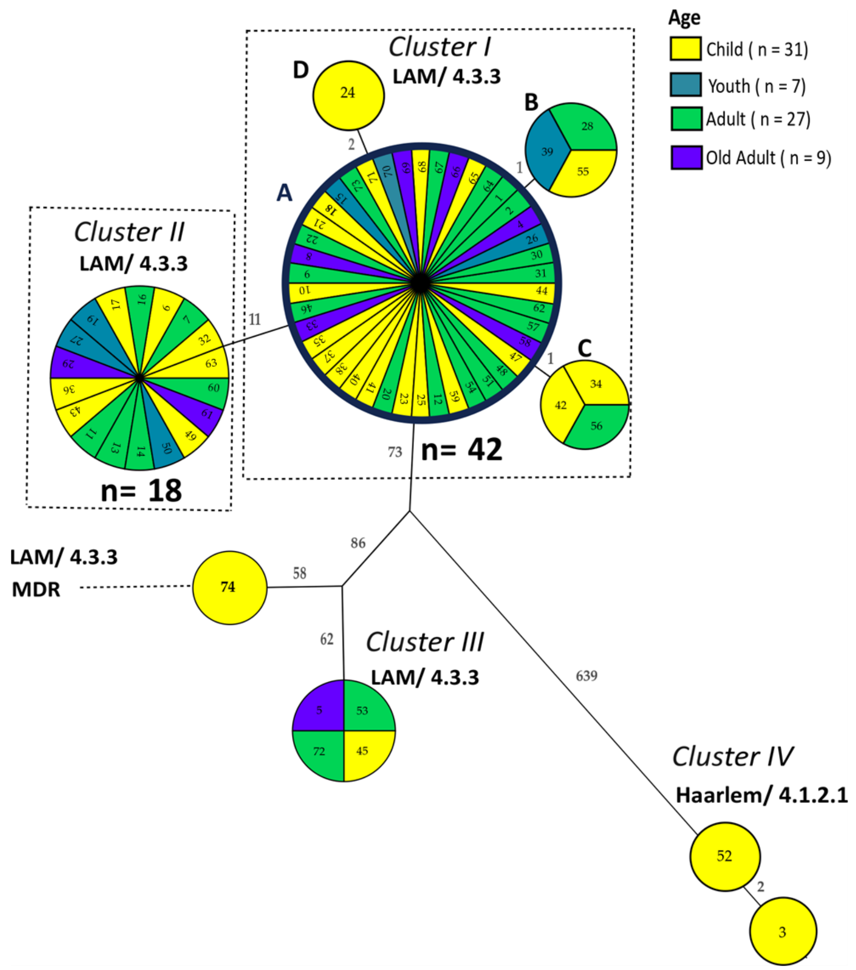

FIG S2 Genetic relationship of patient isolates based on age category. Standard WGS analysis using H37Rv as the reference genome. Maximum Parsimony tree (MPT) based on 890 concatenated Single Nucleotide Polymorphisms (SNP) of 74 *Mycobacterium tuberculosis* (*Mtbc*) isolates from 16 indigenous settlements located on the shore of the Amazon River (AR) or Loretoyaco River (LR). The size of each node is proportional to the number of isolates. The genetic distance is indicated on branches as the number of SNPs that differ between nodes. Samples are color-coded based on age category (Child, < 15 years old; Youth, 16-26 years old; Adult, 27-59 years old; Old Adult, ≥ 60 years old). Outbreak clusters are named I, II, III, and IV. Clusters comprising the clonal events are shown with dashed boxes, and cluster I nodes were termed A, B, C, and D. Abbreviations: LAM, Latin American Mediterranean; MDR, Multidrug-resistant.

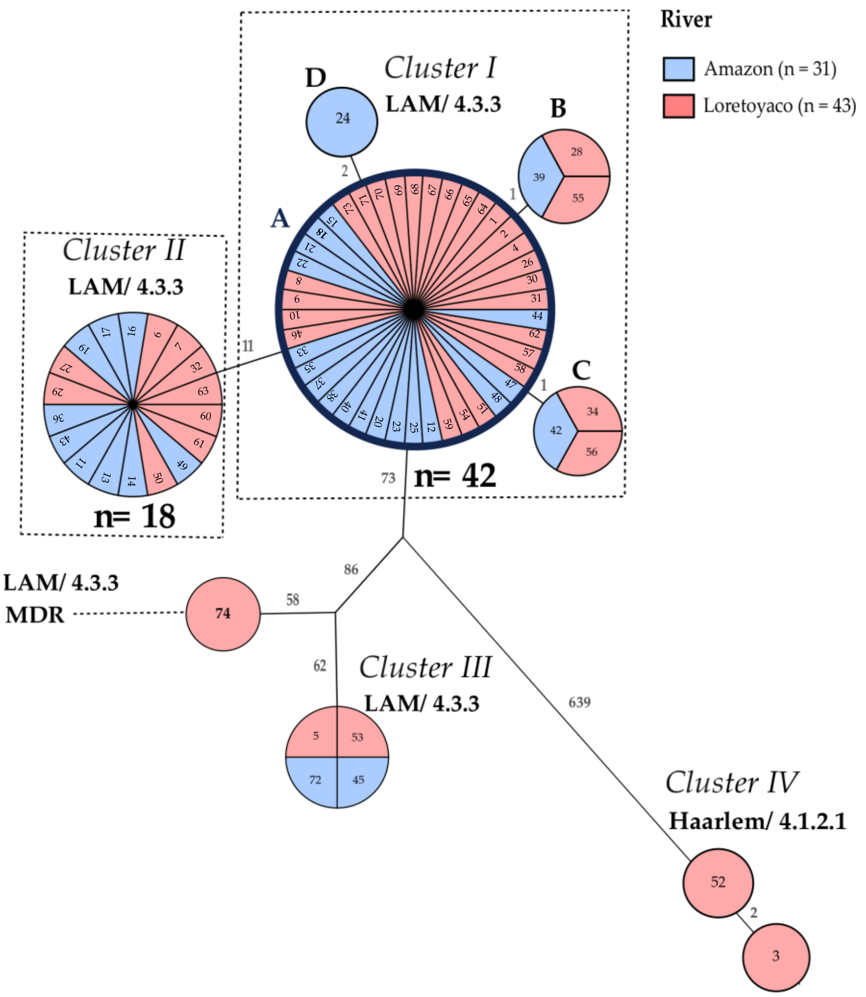

FIG S3 Genetic relationship of patient isolates based on the river location. Standard WGS analysis using H37Rv as the reference genome. Maximum Parsimony tree (MPT) based on 890 concatenated Single Nucleotide Polymorphisms (SNP) of 74 *Mycobacterium tuberculosis* (*Mtbc*) isolates from 16 indigenous settlements located on the shore of the Amazon River (AR) or Loretoyaco River (LR). The size of each node is proportional to the number of isolates. The genetic distance is indicated on branches as the number of SNPs that differ between nodes. Samples are color-coded based on the patient 'river location Outbreak clusters are named I, II, III, IV. Clusters comprising the clonal events are shown with dashed boxes, and cluster I nodes were termed A, B, C, and D. Abbreviations: LAM, Latin American Mediterranean; MDR, Multidrug-resistant.

TABLE S2 Comparison of COL-2 outbreak genome and H37Rv reference genome.

Locus ID	COL-2	H37Rv	Gene ID	Gene name	Annotation
**NP_216037.1**	LOF_DEL		Rv1520	*Rv1520*	Sugar transferase
**NP_216036.3**	LOF_DEL		Rv1521	*fadD25*	Fatty-acid-AMP synthetase (fatty-acid-AMP synthase)
**NP_214891.1**	LOF_DEL		Rv0377	*Rv0377*	HTH-type transcriptional regulator
**NP_216867.1**	new CDS	*INS* - *mobile element*	Rv2351c	*plcA*	Membrane-associated phospholipase C 1 PlcA (MTP40 antigen)
**NP_215071.1**	new CDS		Rv0557	*mgtA*	Mannosyltransferase MgtA
**MF_01206**	new CDS		NA	*msrP*	Rotein-methionine-sulfoxide reductase catalytic subunit MsrP
**NP_215190.1**	new CDS		Rv0676c	*mmpL5*	Probable conserved transmembrane transport protein MmpL5
**NP_216411.1**	LOF		Rv1895	*Rv1895*	Zinc-binding alcohol dehydrogenase
**NP_216793.1**	new CDS	*INS* - *mobile element*	Rv2277c	*Rv2277c*	Glycerolphosphodiesterase
**NP_214568.1**	new CDS	*INS* - *mobile element*	Rv0054	*ssb*	Single-strand DNA-binding protein
**NP_217152.1**	LOF_INS		Rv2636	*Rv2636*	O-phosphotransferase
**NP_217629.1**	LOF_INS		Rv3113	*Rv3113*	Phosphatase
**YP_177926.1**	new CDS	*INS* - *mobile element*	Rv3110	*moaB1*	Pterin-4-alpha-carbinolamine dehydratase
**YP_177925.1**	new CDS		Rv3109	*moaA1*	Cyclic pyranopterin monophosphate synthase
**NP_215783.1**	new CDS		Rv1267c	*embR*	Transcriptional regulator EmbR
**NP_217990.1**	new CDS	*INS* - *mobile element*	Rv3473c	*bpoA*	Peroxidase BpoA

Abbreviations: LOF, putative loss of function; CDS, coding sequence; INS, insertion; DEL, deletion
